# Hypotheses concerning structuring of extruded meat analogs

**DOI:** 10.1016/j.crfs.2023.100510

**Published:** 2023-05-24

**Authors:** R.G.M. van der Sman, A.J. van der Goot

**Affiliations:** a Wageningen Food Biobased Research, the Netherlands; b Food Process Engineering, Wageningen University, the Netherlands

**Keywords:** Meat analog, Extrusion, Soft matter physics

## Abstract

In this paper, we review the physicochemical phenomena occurring during the structuring processes in the manufacturing of plant-based meat analogs via high-moisture-extrusion (HME). After the initial discussion on the input materials, we discuss the hypotheses behind the physics of the functional tasks that can be defined for HME. For these hypotheses, we have taken a broader view than only the scientific literature on plant-based meat analogs but incorporated also literature from soft matter physics and patent literature. Many of these hypotheses remain to be proven. Hence, we hope that this overview will inspire researchers to fill the still-open knowledge gaps concerning the multiscale structure of meat analogs.

## Highlights

• Multitude of new hypotheses are formulated for structuring of extruded meat analogs.
• Structuring of meat analogs occurs at three different length scales.
• Materials resemble of microgels, associative polymers, and transient networks.

## 1 Introduction

In this paper, we review the physicochemical phenomena occurring during high-moisture-extrusion (HME) of plant-based meat analogs. Meat analogs receive a lot of research interest as a tool to achieve a more balanced diet between proteins from animal and plant sources [Aiking and de Boer, 2020], which is a response to societal concerns regarding animal welfare, sustainability, and health. It is thought that the protein transition can be advanced if meat analogs can mimic the eating quality of meat regarding its taste, fibrous and tender texture, and the moist juiciness [Hoek et al., 2004, 2011]. This will allow the consumer not to change their eating habit.

**Fig. 1:**
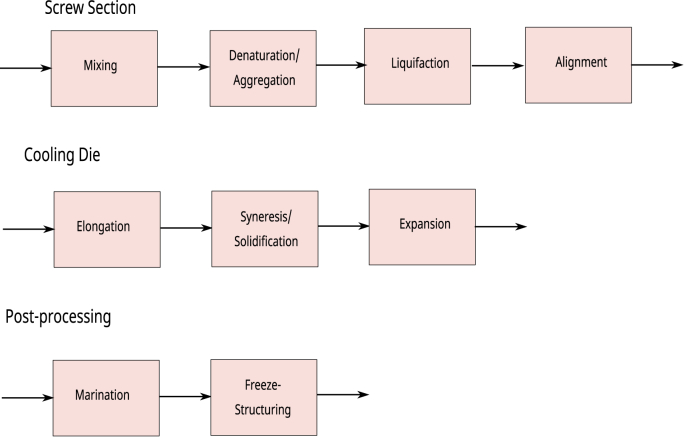
Tasks subsequentially performed during High Moisture Extrusion (HME) of meat analogs, divided over screw section, cooling die, and post-extrusion treatments.

Past scientific research on meat analogs has been primarily technology driven [Pietsch et al., 2019a][Cornet et al., 2021a]. The technology has matured sufficiently, but the current meat analogs still cannot match the quality attributes of meat. We think that to improve the quality of meat analogs, research being driven by product requirements and consumer needs, following ideas of reverse-engineering, or product-driven-process-synthesis is required. [Wibowo and Ng, 2001][Almeida-Rivera et al., 2007][Gupta and Bongers, 2011]. These design methods define food ingredients as input to a process, which must achieve a certain desired output. Our case deals with foods having plant proteins as the primary ingredient, with the desire to manufacture products mimicking muscle meat as close as possible. The process is subdivided into several tasks – which are based on hypotheses regarding physicochemical phenomena happening during the processing of the food materials. These tasks are indicated in Fig. 1.

As also expressed in recent reviews and papers on high moisture meat analog extrusion [Wittek et al., 2021b; Ubbink and Muhialdin, 2022; Schmid et al., 2022; Guyony et al., 2022], we think there is still a limited number of hypotheses on the actual physicochemical phenomena happening during the manufacturing of HME-based meat analogs. The objective of this review paper is therefore to extend this knowledge base of hypotheses and thus to inspire researchers for further investigations to enable the product-driven design of a high moisture extrusion process. We have gathered our hypotheses not only from food science literature and patents on plant protein extrusion but also from other science areas where similar phenomena are happening. We have to note that many of these hypotheses still needs further validation for the case of meat analog processing.

To help the reader to distinguish hypotheses, which are commonly formulated for meat analogs, and those from other fields, we think apply to meat analogs, we decided to underline these hypotheses. Red underlining indicates existing hypotheses, usually commonly accepted for meat analogs, and blue underlining indicates novel hypotheses (from other fields), which we think might apply to meat analogs too.

Following the spirit of product-driven-process-synthesis, we start with the question regarding which input materials are required to achieve the desired output, i.e. plant-based foods resembling fibrous muscle meat. In particular, we focus on the recent pertinent question of how many phases are required to acquire the desired anisotropic fibrous structure. Subsequently, we move forward to assign different tasks to the HME process, based on the main hypotheses of the structuring process. We pose that this structuring happens at three different length scales, but at different locations of the HME process. After stating the different functional tasks of the HME process, we give a detailed account of our understanding of the physical processes at each functional step of the HME process.

## 2 Input material

The requirement for meat analogs is that all ingredients are plant-based, with at least one plant protein. That main protein ingredient is often mixed with another plant protein or a polysaccharide. In practice one often uses protein concentrates, which is less refined than a protein isolate, containing also polysaccharides, mostly originating from cell wall material. These biopolymers are mixed with water at a level between 40 and 80%, but more often between 50 and 60%. Water acts as a plasticizer, modulating rheology and texture. Furthermore, it provides juiciness during eating.

Furthermore, meat analog formulations have other minor ingredients for additional functionality, such as oil, acting as a lubricant and contributing to juiciness, as it stimulates saliva production. As several other review papers have already focussed on the functionality of ingredients [Kyriakopoulou et al., 2021][McClements and Grossmann, 2021], we restrict ourselves to an overview of ingredients and additives, as listed in Table 1. We note, that in the table we distinguish hydrocolloids (added at levels of a few percent) from other polysaccharides, as recent research has shown they appear to have different functionality as polysaccharides as starches or cellulosic fibers, which are added at larger quantities to function as a dispersed phase. Their difference will be explained in more detail below.

**Table 1:** Functional Ingredients applied in high-moisture meat analogs.

Ingredient type	Functionality
Water	Plasticizer, juiciness
Plant protein	Structurant (matrix former), macronutrient
Polysaccharides	macronutrient, water holding, dispersed phase
Hydrocolloids	Structurant, water holding, dietary fibers, binders
Plasticizers	Flavor pre-cursor, plasticizer, reducing agents
Oil/fat	Flavor, tenderness, juiciness, lubricant
Flavoring	Flavor
Colorants	Appearance
Fatty acids (omega)	Nutrition, lubricant
Organic acids	pH adjustment, plasticizer, crosslinker, chelator
Vitamins	Nutrition
Minerals	Nutrition, Crosslinking, pH adjustment

The main source of knowledge on (the functionality of) additives is the patent literature. In the Appendix A, we list several sources used for assembling the table. Some ingredients have multiple roles, like flavour precursors as amino acids or sugars, that also act as plasticizers. In addition to their functionality as a plasticizer, reducing agents like cysteine or glutathione (also present in yeast extracts) have also the functionality of promoting SS/SH exchange of disulphide bonds.

For the remainder of this section, we only focus on the functional requirements of biopolymer ingredients and their possible universal traits. In particular, we discuss the question of how many different (biopolymer) phases are required to achieve the desired anisotropic, fibrous microstructure of meat analogs.

### 2.1 Number of biopolymer phases

Commonly, meat analogs produced by HME are made from a blend of two biopolymers, one being a plant protein and the other being another protein or a polysaccharide. This use of biopolymer blends is based on the hypothesis that two immiscible phases are needed for fibrous structure formation [Akdogan, 1999][Cheftel et al., 1992][Yuryev et al., 1990][Tolstoguzov, 1993a]. This hypothesis is also held for meat analogs made with a shear cell [Dekkers et al., 2018a].

However, recent research shows that perhaps a two-phase system (as described above) is not a prerequisite for fiber formation in meat analogs. Anisotropic structures have been obtained after extrusion or shearing for pure gluten [Pietsch et al., 2019b][Cornet et al., 2021b], pea protein isolate [Osen et al., 2014], and soy protein isolate [Wittek et al., 2021c]. On one hand one can view these ingredients as a single protein phase, but on the other hand, these protein isolates can still contain up to 25% non-protein ingredients - warranting them as a two-phase system. Yet, for soy protein isolate, it is shown that a protein-rich and a water-rich phase are created via a phase-separation process [Wittek et al., 2021c], which we think is more akin to syneresis, as we will discuss below in more detail, and where the second biopolymer phase does not play a major role.

Still, we think there is still a functional role of the second biopolymer phase, which might be more important concerning fracturing properties [Teyssandier et al., 2012]. It happens that the fibrous structure of meat analogs is often only observed after tearing or bending of the final product, but not via slicing [Pietsch et al., 2017][Osen et al., 2014, Diaz et al., 2022, Nisov et al., 2022, Schreuders et al., 2022]. During tearing the mechanical stresses will be concentrated at the interfaces of the two immiscible biopolymer phases - which is even more enhanced if the dispersed phase has a low elastic modulus [Buxton and Balazs, 2005]. A similar role as the second biopolymer can be played by another dispersed phase, such as air [Dekkers et al., 2018c][Wang et al., 2019b,c] or oil droplets [Kendler et al., 2021; Wang et al., 2022]. The inclusion of air can be stimulated by another dispersed phase present like maltodextrin [Wang et al., 2019a] or an emulsion [Wang et al., 2022].

A recent trend is to add a hydrocolloid as a third biopolymeric phase [Nanta et al., 2021; Dou et al., 2022; Schreuders et al., 2022; Dinani et al., 2022]. This addition of a strong water-binding hydrocolloids strongly enhances the fiber formation, and overall water holding capacity [Dou et al., 2022; Dinani et al., 2022]. It is likely, that in the case of syneresis of the protein phase, the expelled water will be absorbed by the hydrocolloid phase [Dou et al., 2022].

### 2.2 Proteins

Upon consideration the plant proteins used for meat analogs [Kyriakopoulou et al., 2021][McClements and Grossmann, 2021], we conclude most of them are storage proteins, originating from oilseeds, pulses, and cereals – where they are found in a highly concentrated form of protein bodies [Shevkani et al., 2019]. Furthermore, we observe a remarkable overlap with proteins used for thermoplastics and coatings [Hernandez-Izquierdo and Krochta, 2008][Verbeek and Van Den Berg, 2010], and hence we think that they have universal traits, that make them suitable for coatings and/or meat analogs. To be functional for meat analogs or coatings, the globular storage proteins first have to be unfolded and realigned to form a new three-dimensional network, that is stabilized by new inter- and intra-molecular interactions [Verbeek and Van Den Berg, 2010]. After unfolding all proteins behave more like random-coil polymers [Boire et al., 2019], which then can entangle and form a network. Prolamine proteins like gluten, rich in proline/hydroxy-proline, are more random in their native state and will readily behave like synthetic random-coil polymers during processing [Verbeek and Van Den Berg, 2010]. Food proteins containing more than 10% of proline residues are gluten, gelatin, zein, and casein, which are known for their good film-forming properties.

We note that proteinaceous ingredients used for meat analogs are often in the form of protein isolates, where the proteins already have been denatured via their preprocessing. In literature, it is questioned whether protein isolates can be treated as a single thermodynamic phase, as many protein isolates are still a mixture of different proteins, as in the case of soy [Wittek et al., 2021b]. However, research has shown that some intermolecular bonding (like disulphide bridges) is happening during denaturation in mixtures of proteins, leading to co-aggregation, and thus single-phase behaviour [De Graaf, 2000][Ersch et al., 2015][Lambrecht et al., 2018]. Co-aggregation is not expected, if meat analogs are made with two different proteinaceous sources like soy and gluten [Lambrecht et al., 2018][Zhang et al., 2020b].

### 2.3 Polysaccharides

Polysaccharides have been used as a secondary phase in meat analogs, such as starches or maltodextrins [Akdogan, 1999][Cheftel et al., 1992], and cell wall materials as endogenously present in protein concentrates or flours [Kyriakopoulou et al., 2021][Cornet et al., 2021a]. Little interactions (i.e. intermolecular bonding) between the immiscible polysaccharide and protein phases are expected. The phases will mainly interact via their differences in water binding [Bühler et al., 2021].

Recent studies indicate that it is advantageous to make a distinction between soluble and insoluble fibers, as they show quite different functionalities [Schreuders et al., 2022; Deng et al., 2023; Nieuwland et al., 2023]. Insoluble fibers like cellulose make meat analogs tougher, with a potential for macroscopic phase separation, while soluble fibers like pectin make the meat analog softer or mushier, but also enhance their water binding [Schreuders et al., 2022]. Soluble fibers are also prone to degradation, via the intense shear and temperature [Dekkers et al., 2018c]. This probably explains why mixtures of SPI and soluble fibers shows no structuring upon extrusion [Nieuwland et al., 2023]. Mixtures of SPI and insoluble fibers due show a fibrous structures, albeit with smaller widths of water-rich domains than pure SPI samples [Nieuwland et al., 2023].

For good textural properties, it seems better to have a mixture of both insoluble and soluble fibers [Schreuders et al., 2022], making the meat analog effectively a ternary system. It is also remarked that soluble and insoluble fibers strongly differ in their water holding capacity (WHC), with insoluble fiber having a WHC lower than the protein phase, while soluble fibers having a higher WHC than the protein phase [Deng et al., 2023]. This difference in WHC influences the water distribution, and thus the phase volumes and rheology. For fiber formation it is important that the protein phase remains the continuous phase [Deng et al., 2023]. This condition depends indeed on phase volumes, and their differences in rheology [Onuki, 1994].

Unmodified starches and maltodextrins are likely to behave as soluble fibers [Bühler et al., 2021]. If they retain a particulate nature, enabled via their modification, the starches can play a role as filler or fat replacer, analogous to cheese analogs [Ye and Hewitt, 2009]. But, waxy and gelatinized starches lowered the degree of fibrousness in soy/starch mixtures [Zhang et al., 2016]. Application of crosslinked native starches in cheese analogs shows though, that if starch can retain its colloidal character during extrusion, it functions as an inert filler particle and promotes the separation of protein fibers [Oberg et al., 2015][Butt et al., 2019][Kern et al., 2020].

Strong water-binding hydrocolloids have been explicitly added to meat analogs formulations [Nanta et al., 2021; Dinani et al., 2022; Taghian Dinani et al., 2023]. Alginate has promoted fibrous structure formation in peanut protein [Zhang et al., 2020a]. Patent (WO2018/177717) provides a recipe for a meat analog including a mixture of xanthan gum and galactomannan for enhancement of juiciness. The two hydrocolloids form together a thermoreversible gel, which melts during final cooking, enhancing the juiciness perception by the consumer.

Hydrocolloids are also used for low-fat cheese analogs to promote fiber formation [Dubey, 2011] [Oberg et al., 2015][Dai et al., 2018]. It is shown that these hydrocolloids as a ternary biopolymer phase promote fiber formation and enhance juiciness in meat analogs [Dinani et al., 2022; Taghian Dinani et al., 2023]. Surprisingly, the anisotropy in mechanical strength and microstructure decreased with increasing concentration, with hydrocolloids ranging from 1 to 3%. At 1% concentration, the fibers were long and with a small diameter. At higher concentrations, the fibers become shorter but thicker.

## 3 Structuring hypotheses

Wittek reported two main hypotheses governing the fibrous structure formation in HME [Wittek et al., 2021b]. The first hypothesis is fiber formation via the alignment of protein (aggregates) in strong shear flow beyond denaturation temperatures, which was originally formulated for low-moisture extrusion [Akdogan, 1999].

The second hypothesis is based on the deformation of a second dispersed biopolymeric phase, as suggested by Tolstoguzov [Yuryev et al., 1990][Tolstoguzov, 1993a]. In the same paper, Wittek reported anisotropic structure formation via phase separation in meat analog formulation with only soy protein isolate, which is difficult to explain with either of the above hypotheses. Later, we have shown similar observations for extruded SPI, using X-ray tomography (XRT) of frozen samples [Nieuwland et al., 2023]. XRT shows a layered structure, with ice formed in a water-rich phase, surrounded by a protein-rich phase. Below, we will make the case that this phenomenon is akin to syneresis, and provides a third, alternative mechanism for anisotropic structure formation.

We view that all three above hypotheses are valid mechanisms contributing to the fibrous structure formation of meat analogs, only they operate at different length scales, and consequently at different locations in the extruder [Nieuwland et al., 2023]. Under the right processing conditions, there can be even a fourth length scale for structure formation: pore development at the exit of the cooling die if the exiting product temperature is still above boiling point. The release of pressure upon exit enables the growth of steam bubbles, leading to a moderate increase in porosity.

**Fig. 2:**
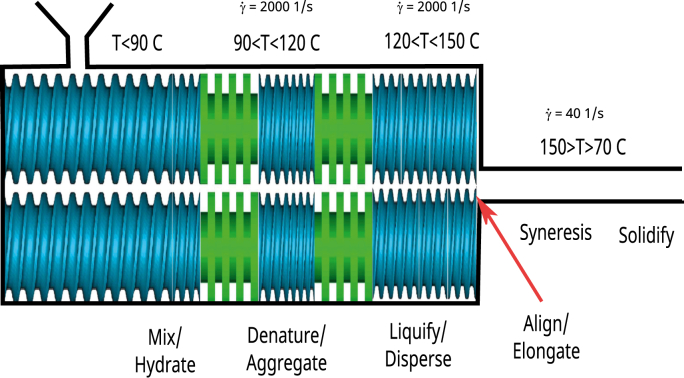
Assignment of tasks to particular sections of the extruder. Estimated temperatures and shear rates are taken from [Cornet et al., 2021a].

Based upon the above hypotheses we have assigned different functional tasks to the HME process, which is a refinement of the tasks defined in our earlier review [Cornet et al., 2021a]. The new division of tasks we have listed in Fig. 1. This subdivision of tasks we will use in our further discussion of hypotheses concerning the physicochemical phenomena happening during HME. As shown in Fig. 2 these functional tasks can be assigned to different sections of the HME process. These locations follow easily from the discussion of physico-chemical processes during extrusion in the next section.

## 4 Physico-chemical phenomena during HME

### 4.1 Mixing

We expect that the total feed undergoes a first dispersive mixing and homogenization at the first constrictive element at a temperature below the melting (denaturation) temperature(s) of the biopolymers [Cheftel et al., 1992][Thiry et al., 2015]. Immiscible biopolymers remain in separate phases, especially during heating as it is expected that heating even increases their incompatibility [Polyakov et al., 1997]. The homogenization of the biopolymer phases is evident in the size reduction of their domains [Kruijt et al., 2001]. The temperature should be below denaturation temperature as the early formation of crosslinks is thought unfavorable for homogenization [Verbeek and Van Den Berg, 2010]. We assume that the dough, entering the melting zone, is a random, isotropic mixture, with small domain sizes - as defined by the thermomechanical conditions in the first constrictive element. Dead-stop experiments show indeed that the dough has a paste-like, somewhat lumpy consistency [Chen et al., 2022; Dou et al., 2022; Zhang et al., 2022].

During mixing we expect (re)distribution of water over the biopolymer phases [Cornet et al., 2021a; Deng et al., 2023]. However, the water distribution can be modulated by the different mechanical stresses on the two biopolymer phases, as imparted by the protein dough structure [Cornet et al., 2020b]. This is observed in biopolymer blends containing gluten [Ooms and Delcour, 2019][Cornet et al., 2020b][Cornet et al., 2021b]. Via shear during mixing gluten is aggregated, stretched, and a network is formed. The network is formed via hydrogen bonds, disulphide bridges, and hydrophobic interactions [Hackenberg et al., 2018]. However, we question whether this gluten network survives during the passage of the kneading elements more downstream of the extruder barrel, as the intensive shear and temperature will degrade large scale structures. But its effect on water redistribution will probably remain, as this is independent of domain size.

For mixtures of proteins and polysaccharides to resemble meat analogs, we assume that a protein should form the continuous phase, otherwise the resulting product will have more resemblance to a starchy snack [Kristiawan et al., 2018]. Rules for deciding what becomes the continuous phase are not fully known yet, but we expect that general rules apply, as given by [Onuki, 1994]. Thus, a low-viscosity component tends to become continuous during mixing to reduce dissipation.

If oil is added to the protein dough, oil droplets need to remain stable to prevent them from coalescing and lubricating the extruder barrel and cooling die walls. As a consequence of this lubricating film, the will be some (apparent) slip of the protein material along the wall, lowering the shear stresses and thus the anisotropic structure development within the protein dough. To mitigate this problem, the oil phase is preferably added as an emulsion. The emulsion needs stabilization with surface active ingredients on their interface, with strong elastic properties, that can survive harsh extrusion conditions. Literature studies of interfacial stability under such conditions are scarce [Sagis, 2011][Sagis and Fischer, 2014]. Large deformation testing gives insight into the yielding behaviour of the interfaces, and this information can be used to design the interface in such a way, that the rate of coalescence during extrusion is greatly reduced.

### 4.2 Denaturation and aggregation

Native proteins unfold above their denaturation temperature, due to increased chain mobility enabled by lowered strength of their intramolecular bonds, which allows them to make intermolecular bonds and form aggregates [Lumry and Eyring, 1954]. Often the proteins in meat analog doughs originate from protein isolates or concentrates, which are already denatured before they are subjected to HME. But gluten is often added as vital gluten (which will thermoset) [Chiang et al., 2019][Maung et al., 2021]. Irrespective of whether the protein is still native or not, we expect sufficiently increased chain mobility above the denaturation/thermosetting temperature. Hence, in the melting zone with 90 < *T* < 120^*o*^*C* the enhanced chain mobility will lead to further aggregate formation. We note that the denaturation temperature is moisture-dependent [van der Sman, 2016].

Due to the diversity of side-groups of proteins, there is a variety of intermolecular bonds possible between proteins, namely hydrogen bonds, hydrophobic interactions, salt bridges, and disulphide bonds. Disulphide bonds are often classified as covalent bonds, because of their moderately high activation energy for bond cleavage. In literature, there is a discussion on the relative importance of disulphide bridges versus other non-covalent bonds [Ledward and Tester, 1994][Morel et al., 2002][Osen, 2017][Liu and Hsieh, 2008]. For non-covalent (hydrophobic) bonds, one has found few changes between starting material and extrudate in the case of protein isolates used as raw ingredients [Osen et al., 2014][Wittek et al., 2021c]. However, one expects significant changes in the number of disulphide (SS) bridges, which are broken in the melting zone of the extruder, as mediated by the so-called SS/SH exchange process [Ledward and Tester, 1994][Morel et al., 2002]. The breaking of SS-bridges is further enhanced by the intense shear in the mixing zone, which is shown to lower the activation energy for S–S bond breakage. Under thermomechanical treatment as in extruders, with the availability of free thiol (SH) groups, the disulphide bridges can be regarded as non-covalent bonds [Lin et al., 2010][Raghavan and Douglas, 2012].

The susceptibility of intermolecular bonds between biopolymers (proteins and polysaccharides) to high temperature combined with high shear stress, as experienced during extrusion, makes us think there is a continuous breaking and forming of intermolecular bonds. Hence, the protein dough can be regarded as a transient network [Redl et al., 1999][Ng et al., 2011]. In the cooling die at sufficiently low temperature, these bonds become more permanent and they can be viewed similarly as crosslinks in a protein gel. The interaction between these (transient) crosslinks and the shear flow determines ultimately the rheology of the protein dough.

In light of the transient network concept, we view the above discussion concerning the relative roles of non-covalent bonds and disulphide bonds is not very relevant. Independent of the type of bond, they all contribute to the density of (transient) crosslinks and thus the rheology of the protein dough. We think it is better to characterize (mixtures of) proteins via rheology, rather than the changes in the different types of bonds the proteins might engage.

The intermolecular bond formation can be modulated by moisture. It is stated that higher moisture content promotes more disulphide bridge formation [Noguchi, 1989], but it also enhances hydrogen bond formation [Chen et al., 2011]. The overall effect is that moisture softens the material [Wu et al., 2019], thus effectively lowering the (transient) crosslink density. Furthermore, water will lower both the denaturation temperature and the glass transition temperature, and thereby also the dough rheology [Bell and Hageman, 1996][De Graaf, 2000][van der Sman, 2016].

The actual denaturation of native proteins is hardly affected by shear [Jaspe and Hagen, 2006], but it promotes aggregation [Sharma and Pandey, 2021]. Reports on extruded whey proteins also conclude that shear does not influence denaturation, but influences both the denaturation and aggregation kinetics via lowering the activation energy [Wolz et al., 2016][Quevedo et al., 2020].

**Fig. 3:**
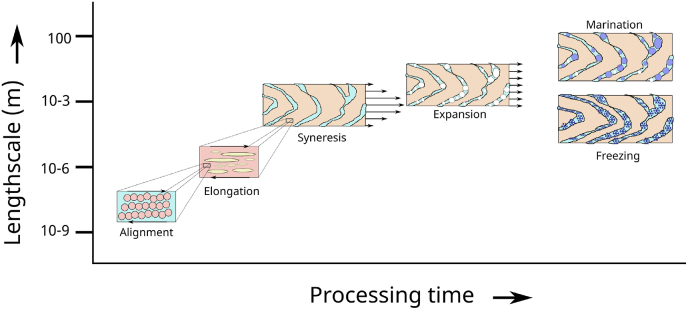
Schematic diagram showing structuring processes happening at different length scales (domain sizes) and different times during processing. Blue colors indicate a water-rich phase, yellow indicates the second biopolymer phase, white indicates air bubbles, dark blue indicated ice crystals, red color indicate protein-rich phase, pink indicates protein aggregate particles. (For interpretation of the references to color in this figure legend, the reader is referred to the Web version of this article.)

Following Smoluchowski's coagulation theory [Wolz et al., 2016] defines several steps in the aggregation process in shear flow: In the first step, there is a growth of aggregates, only driven by thermal fluctuations. In the second step, at sufficient sizes of aggregates, the growth is enhanced by both thermal and hydrodynamic fluctuations, which promote collisions between aggregates, and their further growth. In the third phase, the growth is limited by shear-induced fragmentation of the aggregation. Finally, there is a balance between growth and fragmentation, resulting in a steady particle size distribution. [Badii et al., 2016] show that the application of shear during the gelation of whey leads to stronger and more elastic gel. At higher shear rates even local densification of the gel structure via enhanced aggregation is observed, leading to phase separation of water-rich domains. This growth of protein aggregates we think is happening in the screw section with temperatures in the range 90 < *T* < 120^*o*^*C*, but the shear-induced fragmentation is expected to happen in the next section, where the temperature is controlled in the range 120 < *T* < 150^*o*^*C*, as in this range the intermolecular bonds are sufficiently weakened (as discussed below). These two sections are clearly indicated in Fig. 3.

### 4.3 Liquefaction

For the restructuring of the protein dough it rquires to attain a liquid-like constitution. This process is known as liquefaction, where most of the intermolecular bonds get broken at high temperature and shear conditions [Cheftel et al., 1992]. It is reported that the important intermolecular bonds (hydrophobic and disulphide bonds) between proteins become significantly weak above a critical temperature above 130^*o*^C [Shimada and Cheftel, 1989][Arêas, 1992][Akdogan, 1999]. It is assumed to hold universally for proteins that hydrophobic bonds are broken at *T* > 135^*o*^C [Privalov, 1979; Privalov and Gill, 1988][Tolstoguzov, 1993c]. For the breaking of disulphide bonds, it is probably essential that shear lowers the activation energy for the SS/SH exchange [Pommet et al., 2003].

This liquefaction of the dough is a necessary step for fiber formation. A study on pea-protein isolate shows that no fibrous structure was obtained if dough temperature does not exceed 120^*o*^C [Sandoval Murillo et al., 2019]. At a die inlet temperature of 160^*o*^C, a distinct lamellar structure was observed. Without the liquefaction probably the subsequent processes of alignment and morphology development are not possible. We assume that this liquefaction occurs in the extruder screw section with a temperature within the range 120 < *T* < 150^*o*^*C*.

### 4.4 Alignment

The first structuring process that occurs is at the smallest length scale: the alignment of protein aggregates in the shear flow direction, a process discussed earlier [Purwanti et al., 2010]. For sheared cheese-analogs, this mechanism is shown to happen via small-angle neutron scattering (SANS) [Wang et al., 2019c][Tian et al., 2020].

The experiments show that sheared protein dough remains isotropic on the length scale of single aggregates, whose size is about 10 nm. Anisotropic structures are observed only at length scales of 100 nm and larger, implying that it concerns alignment in the flow direction of aggregates or larger domains only. These elongated structures (fibers) also align along themselves.

After alignment, the aggregates need to be linked. In sheared cheese-analogs, this linkage can be performed via Ca2+-bridges or crosslinking via transglutaminase enzymes [Manski et al., 2007b][Wang et al., 2019c]. The alignment and linkage render strain-hardening properties of the cheese-analogs. In meat analogs, we expect a similar alignment of aggregates, and their linkage is via intermolecular bonds (namely disulphide bridges and hydrophobic interactions), enabled by further cooling of the protein dough in the die section.

Alignment of protein (aggregates) under shear before fiber formation is observed for many natural protein fibers like silk, or slime of the velvet worm [Rössle et al., 2004][Baer et al., 2019][Murphy et al., 2020]. Here, it is said that shear promotes the partial unfolding of the protein monomeric precursors, promoting the assembly of beta structures. This can mean the formation of beta-structures after denaturation/unfolding of proteins present in meat analog doughs can be important for the formation of precursors (aggregates) for fiber formation.

We think the alignment of aggregates has much in common with the mechanisms governing the alignment (filing) of colloids in confined viscoelastic media [Scirocco et al., 2004]. Other authors have made the same connection between the structure of meat analogs, and particle alignment in confined shear fields [Manski et al., 2007a][Tanaka, 2012][Grabowska et al., 2016]. This research gives the important constraint for the rheology of the biopolymer mixture, that it should be shear-thinning. For alignment a critical shear rate needs to be exceeded, depending on viscoelasticity, shear thinning, and particle volume fraction [Pasquino et al., 2013].

This alignment of protein aggregates is probably happening simultaneously with the aggregation and liquefaction processes. A similar combination of processes occurs during the manufacturing of fluid gel/microgels. We think a scaling rule holding for this process also applies to HME, determining the length scale of the protein aggregates.

Carvalho and Djabourov provided a scaling relation for the size of fluid gels, a class of microgels made via biopolymer gelation while shearing [de Carvalho and Djabourov, 1997]. The microgel size is determined by a balance between aggregate growth and breakup, which is expressed by the balance between thermal and mechanical energy. This balance is stated by the Peclet number having the unit value [de Carvalho and Djabourov, 1997][Ghebremedhin et al., 2021]:

(1) $$Pe=\frac{\eta_{\mathrm{eff}}\dot{\gamma }a^{3}}{k_{B}T}\approx 1$$

*a* is the equilibrium size of the aggregate, *η*_*eff*_ the viscosity, $\dot{\gamma }$ the shear rate, *k*_*B*_*T* is the thermal energy, and $\eta_{\mathrm{eff}}\dot{\gamma }$ is the viscous stress. We pose that this balance between thermal energy (*k*_*B*_*T*) and mechanical energy $\eta_{\mathrm{eff}}\dot{\gamma }a^{3}$ also governs the sizing of the protein aggregates in HME. Note, that *η*_*eff*_ can depend on the shear rate, due to the shear-thinning of the protein dough.

This balance, as expressed by *Pe* = 1 will determine the size of protein aggregates, *a*, that can not be broken down further by shear, and they probably will constitute the building blocks of the protein fibers. Consequently, at length scales below *a*, one observes the isotropic molecular structures (of the aggregates), and above this length scale, one can observe the alignment of proteins.

Nicolai discusses the difference between aggregates and microgels [Nicolai and Durand, 2013]. Protein aggregates have a fractal structure, while microgels are more homogeneous, but in the case of dilute suspensions, also hairy particles can be produced [Ghebremedhin et al., 2021]. Fractal aggregates can easily interpenetrate each other. Consequently, the rheology of (non-jammed) suspensions of aggregates and microgels are quite different [Nicolai and Durand, 2013]. However, in quite concentrated suspensions, as in the case of meat analog doughs, one expects only the formation of compact microgels/aggregates. In these dense systems, there is not a clear distinction between microgels and aggregates. [Norton et al., 1999] states that shear provides a unique way of injecting energy into biopolymer blends, differently from thermal fluctuations, promoting different changes of intra/intermolecular associations. Hence, their finding is another indication that the formation, breakup, and alignment of aggregates are governed by the interaction between thermal and hydrodynamic fluctuations at the nanometer scale.

Ronsin expects that microgels suspensions, similar to particulate suspensions, will exhibit shear banding, i.e. development of anisotropy due to the shear [Ronsin et al., 2011]. The microgels form strata, which gradually thicken (due to continued crosslinking) combined with flow localization (=shear banding). This anisotropic structure formation is also observed for fluid gels of kappa-carrageenan, where it is assumed that shear also provides orientation of large coils/helices aggregates. This ordering promotes the shear thinning behaviour [Hilliou and Gonçalves, 2007].

Next to similarities with microgel suspensions, we think that there are also similarities with associative networks, which can form supramolecular structures via transient crosslinks, mediated via hydrogen bonds for example. These transient crosslinks can open up at sufficiently strong shear stresses. Natural materials, like plant cell walls or muscles, able to do self-repair, often show similar behaviour as these synthetic associate polymers [Lalitha Sridhar and Vernerey, 2018]. Protein doughs for meat analogs kind of resemble these associative polymers, as at temperatures above denaturation temperatures, physical bonds like hydrogen bonds, hydrophobic interactions, and disulphide bridges are sufficiently weak to be broken by shear flows. Moreover, the activation energy of disulphide bridges is known to be dependent on the shear stress (see above).

In literature, two mechanisms are described for the shear thickening of associative polymers [Xu et al., 2010]. The first mechanism is strain hardening due to high internal tension in polymers stretched beyond the Gaussian range [Marrucci et al., 1993]. The FENE-P rheological model accounts for that [Yao et al., 2000]. The second mechanism attributes shear thickening due to an increase of (transient) crosslinks [Witten, 1988]. In this picture, intrachain bonds are exchanged for interchain bonds, quite similar to SS-SH exchanges in protein doughs. The increase of interchain bonds builds the extent of an elastic network. Probably in practice both mechanisms for shear thickening play a role [Xu et al., 2010][Thomas Hu, 2014], with an increase of interchain crosslinks happening at short time scales, and non-Gaussian chain stretching at longer time scales.

It is also found that the viscoelastic relaxation times increase with shear stress [Xu et al., 2010], due to the strain hardening (happening at long time scales). Depending on the time scale of bond breaking/formation, the associative polymers can either show strain softening/shear thinning (for fast bond formations) or strain hardening (for slow bond formation processes) [Xu and Craig, 2011]. The maximum shear thickening is happening at a critical shear rate. Beyond that, there is catastrophic network rupture, and the system becomes shear thinning [Lalitha Sridhar and Vernerey, 2018].

Under shear the polymers will first partially break, become stretched and oriented in the flow direction, and subsequently the formation of interchain crosslinks [Mahmoudi et al., 2013][Xu et al., 2010]. The main driver for shear thickening is the shear-induced transformation of intrachain crosslinks to interchain crosslinks [Xu et al., 2010]. The stronger the shear stress, the higher the chance of this transformation. Furthermore, the likelihood of interchain crosslinks perpendicular to the flow direction decreases [Mahmoudi et al., 2013]. Thus, this phenomenon contributes to the formation of filaments of associated polymers in the flow direction, separated by the solution, which is exuded by the enhanced internal stresses in the filaments (due to the enhanced interchain crosslinks). This filamentous structure can show more enhanced shear-thinning behaviour [Mahmoudi et al., 2013].

The filamentous structure is permanent and remains after removal of shear [Mahmoudi et al., 2013]. Hence, the material is showing irreversible thixotropy. The process is also named irreversible shear-induced gelation [Mahmoudi et al., 2013]. For many associate polymers, this structure formation seems reversible [Jin et al., 2013]. It is expected that rigid macromolecules can recover quickly and show reversible structure formation, while flexible macromolecules recover very slowly, or can not recover due to irreversible chain scission [Jin et al., 2013]. If the filamentous structure is irreversibly changed, it will be reflected in the anisotropic elastic properties, as measured with tensile tests [Uchida et al., 2022].

The mechanisms proposed for causing shear thickening for associative polymers, are quite plausible to happen also during the extrusion of meat analog, protein doughs. The high shear breaks bond within protein aggregates, stretch and align them. Upon stretching, the formation of bonds between aggregates/molecules becomes more likely, possibly also driven by the SS/SH exchange (which is sensitive to shear stress). As stated, there is a higher likelihood of bond formation in the shear direction, as opposed to the perpendicular direction. Consequently, a filamentous structure will be formed, as shown by Tian et al., [2020]. In the concentrated systems as protein doughs for meat analogs the proteins molecules have limited flexibility and the recovery processes are slow. Hence, the filamentous structure formation can be irreversible, probably enhanced by the quenching of the recovery in the cooling die.

**Fig. 4:**
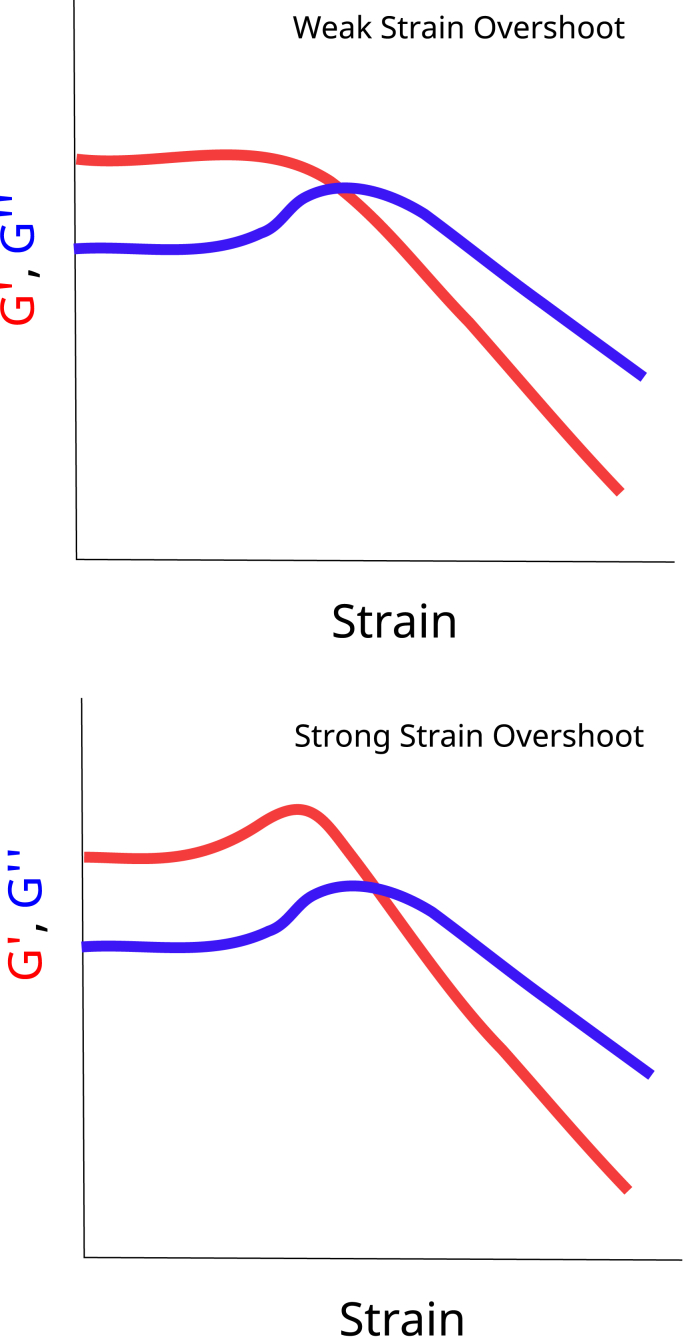
Examples of the interaction between strain hardening and strain softening during Large Strain Oscillatory Sweeps (LAOS), showing weak and strong strain softening, 2 cases of rheological behaviour as posed in the classification of [Hyun et al., 2011].

Mind that this shear thickening process is happening around the critical strain. As discussed above for associative polymers, beyond this critical strain the shear is strong enough to break the bonds, leading to shear thinning/strain softening. Thus, researchers can think this is a paradoxical situation, but it is shown they can occur “simultaneously” in large oscillatory strain cycles, where strain hardening is only happening during part of the cycle, but overall the strain softening is the dominant effect [Mermet-Guyennet et al., 2015]. This interplay between shear thickening and shear thinning is visible in transient networks, as shown by such a transient network model [Sim et al., 2003] and their classification [Hyun et al., 2011]. When performing strain sweeps one observes a maximum in *G*′ and/or *G*″ at the critical strain, which is attributed to shear thickening. Typical responses of large strain sweeps are shown in Fig. 4.

Beyond this critical strain, transient networks show strain softening, which is assumed to have similar causes as shear-thickening [Miyazaki et al., 2006]. The fibrous microstructure formed in meat analogs is assumed to enhance further the shear thinning. Similar effects one observes in polymers with fillers. There, the critical strain depends on the volume fraction of fillers [Haghtalab and Marzban, 2011]. During the extrusion of meat analogs, there is first a breakdown process of aggregates. During the formation of the microfibrous structure there will be a mixture of small and large aggregates, where the large aggregates will function as filler particles. In this process of aggregate breakdown, the critical strain will depend on the number of large aggregates still present, which eventually will be broken down. We think the protein dough transforms from a strong shear thickening to a weak shear thickening material. We have observed such weak strain thickening behaviour in a recent study on the rheology of proteins present in meat analogs [van der Sman et al., 2022].

### 4.5 Elongation of dispersed phase

The second structuring process in HME is the break-up and elongation of the second dispersed biopolymeric phase, as proposed by [Tolstoguzov, 1993b][Dekkers et al., 2018a,c][Wittek et al., 2021a]. [Tolstoguzov, 1993b] states that fiber development requires alignment and deformation of a dispersed phase, with subsequent coalescence into an elongated structure. The alignment and deformation are promoted by the elongational and shear flow in the screw section and the cooling die [Bouvier et al., 2003]. Elongational flow is particularly strong in the transition from the screw section and the cooling die [Meijer et al., 1988].

This structure development is often compared to that of (water-in-water) emulsions [Tolstoguzov, 1993b], similar to the morphology development of (synthetic) polymer blends [Sundararaj and Macosko, 1995]. If the volume fraction of one of the phases is significantly smaller, the minor phase will form droplet-like domains – which will undergo break-up and coalescence events due to the intensive shearing in the extruder. Rapid equilibrium between break-up and coalescence is expected.

Droplet breakup is governed by the viscosity ratio between phases, and the capillary number (Ca), the ratio between Laplace pressure and shear stress, as expressed in the Grace curve [Grace, 1982]. The Grace curve still holds for shear-thinning materials, by taking the shear-rate dependent viscosity [Desse et al., 2011]. Coalescence in extruder mixing is little investigated. Nevertheless, it has been shown [Fortelny and Kovar, 1988] that two rules still apply: 1) there is little coalescence if matrix viscosity exceeds a critical value, which is dependent on the dispersed phase volume fraction, and 2) coalescence increases with a higher dispersed phase ratio, leading to more elongation of dispersed phase [Landreau et al., 2009]. [Delamare and Vergnes, 1996] have developed a model for describing morphology development in a twin extruder using rules for dispersed phase break-up and coalescence. At the percolation threshold, a bicontinuous morphology is formed. Rules for bicontinuous structures are given by [Onuki, 1994], which depend on the ratio of the phases, and their viscosity ratio. [Meijer et al., 1988] states that fibrous structure formation happens if the dispersed phase has a low elastic modulus.

In contrast to emulsions, protein doughs have some elasticity, which also affects the morphology development of the dispersed phase. This interplay between viscoelasticity and morphology development is described by the theory of viscoelastic phase separation (VPS) [Meng et al., 2016][Koyama and Tanaka, 2018]. VPS is expected to occur during the shearing of protein mixtures for meat analogs [Tanaka, 2012]. Special modes of VPS occur if there is an asymmetry in mechanical properties between biopolymer phases, leading to the development of a transient network [Tanaka, 2009]. Asymmetry in mechanical properties is also said to be of importance for meat analogs [Dekkers et al., 2018c]. [Banc et al., 2019] have shown that VPS is occurring in gluten protein mixtures. They show that the VPS in gluten can be arrested via temperature-quenching, i.e. quick cooling.

To enhance the elongational flow it is suggested to place breaker plates in the transition zone between the screw section and cooling die [Cheftel et al., 1992]. Breaker plates are orifice plates with multiple holes, which originate from single screw extruders to sieve out large particulates and provide a more uniform pressure distribution [Harper, 1979][Luxenburg et al., 1985][Ajita and Jha, 2017]. It is also stated that breaker plates can aid in the alignment process of protein aggregates [Akdogan, 1999][Cornet et al., 2021a].

The structuring role of the dispersed phase can also be provided by other phases, such as air bubbles [Tian et al., 2018a][Wang et al., 2019b] or fat globules [Kern et al., 2020]. The air bubbles originate probably from the air included in protein particles produced via spray drying. However, the presence of air is not a prerequisite for fiber formation, but it appears to enhance it [Wang et al., 2019b,c]. Air incorporation is promoted via the addition of maltodextrin. But there is an optimal volume fraction of about 5%. Too much maltodextrin promotes coalescence and shear-induced migration, and thus unwanted phase separation at the macroscale. (We note, that this migration and phase separation we view as indicators that VPS-like processes are happening). Studies on the extrusion of cheese-analogs show that fat globules can also function as dispersed phases, enhancing the formation of a fibrous texture [Kern et al., 2020]. Similar fiber formation also happens in extruded cheese if a polysaccharide (like xanthan or xanthan/guar mixtures) is added as fat replacers [Oberg et al., 2015].

### 4.6 Other reactions during extrusion

The intense thermomechanical treatment as occurs in HME can enhance various physicochemical changes [Verbeek and Van Den Berg, 2010], such as cross-linking, aggregation, SS/SH exchange, fragmentation, chain scission, oxidation, and Maillard reactions [Bekard et al., 2011]. In the last decade influence of thermomechanical treatment on chemistry has evolved into a field of science on its own, named mechanochemistry [Baldus, 2013, Beyer and Clausen-Schaumann, 2005, Gavrilova et al., 2019; Kaupp, 2009; Makarov, 2016; Zhao et al., 2011]. We view these novel insights from these other fields are highly relevant to the extrusion of plant proteins. Several of the physicochemical changes have been discussed already above. Below, we will discuss the changes due to fragmentation/chain scission, Maillard reactions, and oxidation.

Several biopolymers are prone to degradation/chain scission under intense shear and heat, such as starch [van den Einde et al., 2005][Jebalia et al., 2019] pectin [Chen et al., 2014][Dekkers et al., 2016], or whey proteins [Emin et al., 2017]. The length scale of molecules/aggregates is an important parameter for shear degradation, as also indicated by Eq. (1). The smaller size of (globular) protein molecules makes them thus less prone to chain scission, compared to the large-sized starch granules/molecules. The shear degradation of polysaccharides will lower their viscosity, and that will decrease the size of dispersed phases, as expressed by the Grace curve (as discussed above). Furthermore, crosslinking and entanglement enhance the shear degradation [Day and Swanson, 2013].

Maillard reactions may also take place during extrusion, although with a low amount of reducing sugars, high moisture content, short residence time at elevated temperature, and mild extrusion conditions few reaction products were observed [Cheftel et al., 1992]. However, reducing sugars can be made during extrusion via mechanical degradation of polysaccharides like starch or pectin [Koch et al., 2017].

Thermomechanical treatment can also intensify reactions involved in flavour formation, destruction of anti-nutritional factors (ANF), and lipid oxidation [Khan et al., 2015][Nikmaram et al., 2017]. (It must be said that ANF are often already extracted from protein isolates and concentrates). High-moisture and high-temperature conditions can increase the hydrolysis of lipids, which increases potential interactions with the (sulphurous) side chains of amino acids in proteins [Schaich and Rebello, 1999][Tran et al., 2008].

### 4.7 Solidification and syneresis

The anisotropic structure of the protein dough can only be preserved if the structured fluid mass is gelled (solidified), which occurs in the cooling die. From the viewpoint of transient networks, this solidification must be viewed as the rate of intermolecular bond formation is larger than the rate of breakdown. Effectively, the crosslink density increases during cooling, which will be reflected in the rheology by an increase in elastic modulus and viscosity. Our recent studies show that the elastic modulus of several protein doughs is a function of *T*_*g*_/*T* [van der Sman et al., 2022].

This increase in elastic modulus is the cause, we think, of the third structuring process: syneresis. Recently, it has been observed that in the cooling die water-rich and protein-rich domains are formed using purely soy protein isolate (SPI) [Wittek et al., 2021a][Nieuwland et al., 2023]. [Sandoval Murillo et al., 2019] claims this phase separation is due to spinodal phase separation induced by the cooling of the extrudate. We view that spinodal decomposition is not likely to happen as biopolymers in the protein dough do not have a critical state at high temperatures, where they are miscible. It is even assumed that their incompatibility increases with temperature [Tolstoguzov, 1993c]. For a single protein phase, like SPI, phase separation between a dense protein phase and a water phase can happen during the formation of coarse gels [Leksrisompong et al., 2012]. However, this phase separation is not via spinodal decomposition. We think this phase separation is due to crosslinking happening during solidification. As explained by Flory-Rehner theory [Cornet et al., 2020a] an increase in crosslinking lowers the water-holding capacity of proteins. If the excess water can not be absorbed by another biopolymeric phase (like a polysaccharide), the excess water will form a separate phase.

Similar physics is happening during the syneresis of cheese rennet [Walstra, 1993][Fagan et al., 2017]. Phase separation is observed also during the extrusion of mozzarella cheese [Kern et al., 2020]. It is thought to be similar to phase separation happening during meat analog extrusion. Phase separation in the extrusion of cheese is explained by aggregation that happens via hydrophobic bond formation between protein aggregates, leading to serum release. Syneresis is also reported to happen in cheese-analogs undergoing crosslinking during shearing [Manski et al., 2007b, 2008]. If residence time in the shear field is too long, there is too much crosslinking, leading to fracturing (or formation of a granular structure) and separation of serum. Hence, for meat analogs, we also expect significant interactions between 1) the changes in rheology due to crosslinking (transient bond formation), 2) the resulting gradients in shear stresses, and 3) the water distribution between phases. Moreover, we think much of the physics in this stage of the HME process is captured by models similar to viscoelastic phase separation [Tanaka, 2012].

An example of the effect of water migration on flow and rheology is shown for a flowing microgel suspension, where serum can flow out of microgel particles, to allow them to pass each other in shear flow [Adams et al., 2004][Desse et al., 2011; Zeo et al., 2005]. These microgels show deformation and breakup, similar to emulsion droplets. We view the protein aggregates making up the meat analog dough can be viewed as colloidal-sized microgels.

The formation of a water-rich domain is recently observed via XRT for an extruded sample of pure SPI [Nieuwland et al., 2023]. Ice crystals nucleate in these water-rich domains, and the final structure of the frozen meat analogs resembles the wedge shapes obtained after pulling unfrozen samples. This structure is the V-shaped macroscale structure as indicated in Fig. 3. Surprisingly, the XRT images show there is some interaction between the syneresis process and the presence of the second biopolymer phase, which can change the thickness of the V-shaped lamella. Soluble and insoluble fibers, as present in SPC, have different effects on the macroscale structure. Moreover, the presence of the second biopolymer phase made the fracturing of the samples during pulling much easier. SPI samples were nearly impossible to pull apart [Nieuwland et al., 2023]. These observations align with the above hypothesis that the second biopolymer phase promotes fracturing during eating.

Solidification will take place if the viscous stress drops below the yield stress [Högg et al., 2017]. Hence, plug flow can develop in the middle of the die, with a strong velocity gradient along the wall. Due to the strong coupling between rheology and temperature (via *T*_*g*_/*T*) it is suggested that the shape of the temperature profile in the cooling die determines the macroscopic layered structure of the meat analog [Sandoval Murillo et al., 2019]. The similarity between the lamellar structure and temperature profile is also observed for extruded cheese-analogs [Kern et al., 2020].

It is suggested that significant slip will occur during HME [Högg et al., 2017]. It is likely provided by the phase-separated serum that migrates to the wall of the cooling die, providing lubrication and allowing plug flow for the solidified dough. If there is insufficient lubrication slip-stick phenomena can occur, leading to surface defects like shark-skinning or folding, as observed during extrusion of mozzarella cheese [Muliawan and Hatzikiriakos, 2008][Kern et al., 2020].

### 4.8 Expansion

Another way of structuring meat analogs is via expansion of vapor bubbles upon exit of the cooling die. This expansion is commonly happening during low-moisture extrusion of meat analogs, where it is induced if the protein dough temperature at the exit is exceeding boiling temperature. Expansion is the result of the balance between gas overpressure, i.e. vapor pressure minus ambient pressure, and viscous stress [Kristiawan et al., 2019]. For HME the protein matrix is likely to behave as a viscoelastic material, with contributions of viscous and elastic stress, counteracting the gas overpressure. The expansion will start in pre-existing pores present in the protein matrix, incorporated during mixing in the screw section or present in spray-dried protein isolates [Tian et al., 2018b]. This porous structure can enhance the marination of the meat analog, or enhance the juiciness experienced during the first bite [Cornet et al., 2021c].

Another strategy to create pores in the protein dough is via flow instabilities, as described in patent US20200323238A1. It provides a complex cooling die design, transforming the circular cross-section to a wide and thin slit shape. Wells in the walls provide a periodical flow instability in the viscoelastic protein dough. The flow instability induced wide gaps in the protein dough exiting the cooling die. These gaps are provided to enable the dough to be filled with a fat phase or fat-analog. Periodical flow instability provides pressure oscillations, as discussed in more detail in the review [Agassant et al., 2006].

### 4.9 Marination

After extrusion meat analogs can be marinated to deliver flavour or adjust the water-holding capacity/juiciness [Yusop et al., 2010]. Also, the created pores can be filled with fat or fat-analogs, providing a marbling effect as present in certain types of muscle meat, as proposed in US20200323238A1. Some flavours are oil-soluble, and therefore the impregnation fluid can be an emulsion. Via adjusting the ionic strength and pH of the impregnation fluid/marinade the WHC of the protein matrix can be enhanced [Cornet et al., 2021c]. Meat marinade is often rich in NaCl, which is known to solubilize myosin and promote swelling of meat [Sharedeh et al., 2015]. However, in plant proteins, NaCl does not have this specific action, but it just increases the ionic strength that in general lowers the WHC of meat analogs [Cornet et al., 2021c].

Impregnation via simple immersion of non-porous, large pieces is a very slow process driven by diffusion [Harkouss et al., 2018]. Consequently, directly after extrusion the protein dough is often cut or torn into pieces to decrease size and impregnation time. If the meat analog has increased porosity, via expansion, for example, the impregnation can be significantly enhanced via vacuum impregnation [Chiralt et al., 2001]. Via the pressure drop during the evacuation, the gas is drawn out of the meat and replaced by the immersion fluid during depressurization. The vacuum impregnation is particularly efficient if the food material is elastic, which generally holds for meat analogs [Cornet et al., 2021c].

### 4.10 Freeze structuring

The primary function of freezing is of course to render a shelf-stable product during storage. However, freezing can also be used to improve the structure of meat analogs [Yuliarti et al., 2021]. Traditionally in Japan freezing is used to structure soy products, like kori-tofu [Miyawaki et al., 1992]. Ice crystal spacing and thus the fibrous structure can be controlled via freezing rate [van der Sman, 2013]. A good fibrous structure can be obtained for soy-based scaffolds if directional freezing is applied, where the ice crystals (dendrites) grow in the direction of the temperature gradient [Guan et al., 2010]. Freezing tends to promote further crosslinking/aggregation of protein network [Yuliarti et al., 2021] leaving a capillary water phase after thawing [Kalichevsky-Dong et al., 2000][Hernández et al., 2004][Lozinsky et al., 2008]. It is expected that the formation of additional crosslinking is also dependent on the freezing rate [Harnkarnsujarit et al., 2016]. We assume that the ice crystal formation starts in the water-rich phase, which is created via syneresis happening during solidification, as the elasticity of the protein-rich phase hinders ice nucleation there. The ice growth will enhance the growth of the water-rich phase, at the expense of the protein-rich phase. In effect, the freezing amplifies the fibrous structure formation initiated by the syneresis during the solidification of the dough in the cooling die. Developments in freeze-casting of biomaterials, such as tissue scaffolds, show that this freeze-structuring can be improved via directional freezing, which promotes ice crystal growth in the temperature gradient direction. The ice crystal growth will enhance the anisotropy of the meat analog structure [Li et al., 2012][Christiansen et al., 2020].

## 5 Conclusions

In this review paper, we have posed that the structuring of meat analogs is happening at different length scales and at different times in the whole HME processing. Our proposition of the multiscale structuring is based on a multitude of hypotheses concerning the physicochemical processes happening at different stages of the HME process, which may include several postprocessing steps like freezing. For the reader's convenience, we have summarized the main hypotheses in Table 2. It must be said that many of the stated hypotheses have still to be validated, but we hope that this overview inspires researchers to investigate that experimentally. This probably requires probing the multiscale structure with different techniques targetting different length scales, and these techniques are just getting used in the field of food science [van der Sman and van Duynhoven, 2022]. In a separate Appendix B we will give some suggestions for experimental analysis of the main hypotheses from Table 2. How to fine-tune the multiscale structure needs knowledge of how all different physicochemical phenomena are coupled to each other, which calls for the development of multiscale simulation models, which are also currently under development in food science [Ho et al., 2013][van der Sman, 2022].

**Table 2:** Main hypotheses concerning meat analog structuring in extruders.

1	Structure formation occurs at three length scales:
	a) protein aggregate alignment at the nanoscale,
	b) second biopolymer phase elongation at the microscale, and
	c) phase separation of water via syneresis at mm scale.
2	For structuring a second biopolymer phase is not per se required, as a single protein phase already exhibits syneresis. But, the additional second biopolymer phase will improve texture via enhancing fracturing during eating.
3	Size of protein aggregate/fibrils at the nanoscale is controlled by balance between mechanical (shear) energy and thermal energy.
4	Elongation of the dispersed phase is governed by shear and elongational flow, following common rules for emulsions and biopolymer blends.
	This mainly happens in the transition zone between the screw section and the cooling die.
5	In the cooling die syneresis is induced by an increase in elasticity, due to a lowering of temperature, leading to a decrease in water holding capacity and subsequent expulsion of water.
6	Thermomechanical treatment above 120^*o*^*C* induces significant weakening of SS-bridges, and other non-covalent bonds between protein molecules, required for the alignment of aggregates into fibers.

## CRediT authorship contribution statement

**R.G.M. van der Sman:** Conceptualization, Writing – original draft, Writing – review & editing. **A.J. van der Goot:** Writing – review & editing.

## Declaration of competing interest

We declare there are no relevant financial or non-financial competing interests to report, except for the stated funding.

## Figures and Tables

**Table 3:** Functionality of additives in meat analogs from patent literature

Ingredient	Patent	Functionality
cysteine, glutathione	US2009/0208612	reducing agents promoting SS-SH exchange
betaine/sugars/amino acids	US2008/0268112	color change during heating
leghemoglobin/sodium hydrosulphite	AU2018256629	color change during heating
omega-3 fatty-acids	US9526267	health-promoting compounds or micronutrients
	US952626B2	
Leghemoglobin	AU2018256629	micronutrient containing Fe2+
Minerals	AU2018256629	Ca^2+^, Mg^2+^ mineral nutrients
Antioxidants	US952626B2	protecting against lipid oxidation
Vitamins	WO2000/069276	antioxidant/micronutrient
calcium bicarbonate	US9526267	pH adjusting agents/leavening agent
calcium phosphate	US2009/0208612	Acidulent for leavening agent
Ca^2+^	US9526267	crosslinking agent/modulator of protein charge
TGase	EP118297	crosslinking agents
Polyphenols	WO2000/069276	crosslinking agents
citric acid	US2019/0037893	chelators for modulating action of Ca^2+^

## Data Availability

No data was used for the research described in the article.

## Appendix A Ingredient functionality from patent literature

Oil or fat is added to food products to 1) enhance juiciness, as it stimulates saliva production (US2019/0037893, 2019). Furthermore, and 2) to mimic the marbling effect as present in certain meat cuts. As the meat analog is intended to be plant-based, the oils and fats need to be sourced from plants too. Major plant sources are seeds like soy, sunflower, rapeseed, canola, corn, palm, coconut, and sesame (EP3270716, 2015). Retaining the oils in the form of natural oil bodies can provide the required physical and chemical stability (AU2018256629).

A nice classification of additional additives can be found in US9877498. The list in table 3 below follows more or less this classification.

## Appendix B Suggested analysis of main hypotheses

For our main hypotheses, as listed in Table 2, we will suggest several experimental methods for detailed exploration of the validity of the hypotheses.

The first hypotheses concerns the multiscale structuring, which requires complementary (multiscale) experimental methods. The size of protein aggregate buidling blocks, and the anisotropy of protein fibers at nanoscale can be investigated by applying scattering techniques like (U)SAXS, or (U)SANS to extrudates [Manski et al., 2007a; Tian et al., 2020]. The visualization of a second polysaccharide phase is often done via CSLM of extrudates [Dekkers et al., 2018b], where protein and polysaccharide phase or labelled differently. The water-rich phase as induced by syneresis can be imaged via XRT of frozen extrudates [Wittek et al., 2021b; Nieuwland et al., 2023], or via time-domain NMR, where spatial maps of *T*_2_ relaxation times are created, showing domains with different water mobility - indicating domains with free and bound water [Chen et al., 2010]. The XRT methods have already validated the second hypotheses, showing syneresis in pure SPI extrudates [Wittek et al., 2021b; Nieuwland et al., 2023].

The third hypothesis about the length scale of the protein aggregate buidling blocks can be achieved via the scattering techniques [Manski et al., 2007a; Tian et al., 2020], while using extrudates manufactured at different process settings, i.e. ratios of thermal and mechanical energy. The transition between isotropic structure and anisotropic structure will indicate the aggregate length scale [Tian et al., 2020].

The fourth hypotheses requires dead-stop extrusion trials, cf. [Wittek et al., 2021b], and performing CSLM microscopy of samples from different locations. Similar dead-stop experiments can be performed for validation of the fifth hypotheses, combined with analysis using XRT of extrudates [Wittek et al., 2021b; Nieuwland et al., 2023], which can show the location of syneresis happening. If lab-extruders are used, temperature probes can be inserted along the length of the cooling die, getting a measure of the temperature where syneresis is happening. One can easily vary the temperature in different trials in cooling die via use of different coolant temperatures.

The sixth hypotheses has been shown at lower temperatures for wheat doughs [Domenek et al., 2002]. Similar methods can be applied at elevated temperatures, but tempeatures above 100^*o*^C can pose some challenges as water can boil off.
